# Spatial Variation and Land Use Regression Modeling of the Oxidative Potential of Fine Particles

**DOI:** 10.1289/ehp.1408916

**Published:** 2015-04-03

**Authors:** Aileen Yang, Meng Wang, Marloes Eeftens, Rob Beelen, Evi Dons, Daan L.A.C. Leseman, Bert Brunekreef, Flemming R. Cassee, Nicole A.H. Janssen, Gerard Hoek

**Affiliations:** 1 National Institute for Public Health and the Environment (RIVM), Bilthoven, the Netherlands; 2 Institute for Risk Assessment Sciences, Utrecht University, Utrecht, the Netherlands; 3 Department of Epidemiology and Public Health, Swiss Tropical and Public Health Institute, Basel, Switzerland; 4 University of Basel, Basel, Switzerland; 5 Flemish Institute for Technological Research (VITO-MRG), Environmental Risk and Health Unit, Mol, Belgium; 6 Julius Center for Health Sciences and Primary Care, University Medical Center Utrecht, Utrecht, the Netherlands

## Abstract

**Background:**

Oxidative potential (OP) has been suggested to be a more health-relevant metric than particulate matter (PM) mass. Land use regression (LUR) models can estimate long-term exposure to air pollution in epidemiological studies, but few have been developed for OP.

**Objectives:**

We aimed to characterize the spatial contrasts of two OP methods and to develop and evaluate LUR models to assess long-term exposure to the OP of PM_2.5_.

**Methods:**

Three 2-week PM_2.5_ samples were collected at 10 regional background, 12 urban background, and 18 street sites spread over the Netherlands/Belgium in 1 year and analyzed for OP using electron spin resonance (OP^ESR^) and dithiothreitol (OP^DTT^). LUR models were developed using temporally adjusted annual averages and a range of land-use and traffic-related GIS variables.

**Results:**

Street/urban background site ratio was 1.2 for OP^DTT^ and 1.4 for OP^ESR^, whereas regional/urban background ratio was 0.8 for both. OP^ESR^ correlated moderately with OP^DTT^ (*R*^2^ = 0.35). The LUR models included estimated regional background OP, local traffic, and large-scale urbanity with explained variance (*R*^2^) of 0.60 for OP^DTT^ and 0.67 for OP^ESR^. OP^DTT^ and OP^ESR^ model predictions were moderately correlated (*R*^2^ = 0.44). OP model predictions were moderately to highly correlated with predictions from a previously published PM_2.5_ model (*R*^2^ = 0.37–0.52), and highly correlated with predictions from previously published models of traffic components (*R*^2^ > 0.50).

**Conclusion:**

LUR models explained a large fraction of the spatial variation of the two OP metrics. The moderate correlations among the predictions of OP^DTT^, OP^ESR^, and PM_2.5_ models offer the potential to investigate which metric is the strongest predictor of health effects.

**Citation:**

Yang A, Wang M, Eeftens M, Beelen R, Dons E, Leseman DL, Brunekreef B, Cassee FR, Janssen NA, Hoek G. 2015. Spatial variation and land use regression modeling of the oxidative potential of fine particles. Environ Health Perspect 123:1187–1192; http://dx.doi.org/10.1289/ehp.1408916

## Introduction

The associations between long-term exposure to ambient particulate matter (PM) and various adverse health effects have been documented extensively by numerous epidemiological and toxicological studies (Brunekreef and Holgate 2002; Hoek et al. 2013; World Health Organization 2013). Oxidative stress—triggered by the formation of reactive oxygen species (ROS) when PM interacts with cells—has been considered one of the underlying biological mechanisms behind PM-associated health effects (Nel 2005). As a result, suggestions have been made to use oxidative potential (OP) as an additional metric to PM mass concentrations to measure PM toxicity (Ayres et al. 2008; Borm et al. 2007). OP is an intrinsic measure of PM to oxidize target molecules, and thus effectively incorporates biologically relevant properties of PM, such as size, surface, and chemical composition. Despite its plausibility, little empirical documentation exists about whether OP predicts health effects better than currently regulated PM characteristics, including mass and composition.

Epidemiological studies use spatial variation to assess long-term health effects of PM, often accounting for the variations of air pollution concentrations within urban areas (Hoek et al. 2008; Jerrett et al. 2005). Land use regression (LUR) models can effectively explain spatial contrasts, by using statistical modeling to analyze associations between measured concentrations at monitoring sites and predictor variables derived from geographic information systems (GIS) (Hoek et al. 2008). Within the framework of the European Study of Cohorts for Air Pollution Effects (ESCAPE; http://www.escapeproject.eu), LUR models have been developed to estimate the spatial variation of the annual mean concentration for various pollutants including PM mass concentration (Eeftens et al. 2012a), elemental composition (de Hoogh et al. 2013), nitrogen dioxide (NO_2_) and nitrogen oxides (NO_x_) (Beelen et al. 2013). These models were used to assess the association between long-term exposure to air pollution and specific health outcomes.

To our knowledge, only one study has assessed the feasibility of modeling OP of PM for use in epidemiological studies of long-term air pollution exposure. Yanosky et al. (2012) developed a LUR model for the OP of PM_10_ for greater London (UK), where OP was measured as the depletion rate of antioxidant-reduced glutathione (GSH) in a model of human respiratory tract lining fluid. We analyzed OP of PM_2.5_ for the Netherlands/Belgium study area within the ESCAPE study. We aimed to characterize the spatial contrasts of two acellular OP methods, which can provide different information regarding the oxidative properties of PM, and to develop and evaluate LUR models for the spatial variation of annual average OP. These OP models will be used to estimate long-term exposure to air pollution in epidemiological studies, and to test empirically whether OP predicts health effects better than commonly used metrics such as PM_2.5_ mass concentration.

## Materials and Methods

*Air sample collection*. The sampling campaign has been described in detail elsewhere (Eeftens et al. 2012b). Briefly, the study included 34 sites spread over the Netherlands and 6 sites in Antwerp, Belgium (see Supplemental Material, Figure S1 and “Description of the sampling site selections”). Three different site types were selected: regional background (*n* = 10), urban background (*n* = 12), and street sites (*n* = 18). Regional background sites were located in small towns. Urban background sites were located in a large urban area. Regional and urban background sites were at least 50 m away from major roads. Street sites were situated at building facades representative for homes, in streets with traffic intensities of ≥ 10,000 vehicles per day. Between February 2009 and February 2010, three 2-week PM measurements were conducted at each site in the spring/fall, summer, and winter months, resulting in a total of 120 samples. The annual average of OP, used for model development, was calculated for each site and adjusted for temporal variability by using measurements collected continuously for 2-week periods over the entire year at a centrally located reference site. For each sampling period, the temporal correction was calculated as the absolute difference of each OP measurement at the reference site and the annual mean at the reference site (Eeftens et al. 2012b).

NO_2_ and NO_x_ were measured with passive samplers using Ogawa badges (Cyrys et al. 2012). PM_2.5_ was sampled with Harvard Impactors on Teflon filters. These samples were also used to measure absorbance and analyzed for elemental composition using energy dispersive X-ray fluorescence (XRF) at Cooper Environmental Services (Portland, OR, USA). A total of 48 elements were measured. A more detailed description of the elemental composition is available in the study by de Hoogh et al. (2013). Until processing, the filters were stored in Petri dishes at 4°C in the dark.

*Oxidative potential*. In order to measure OP, the Teflon filters were extracted with methanol (HPLC grade). The suspensions were equally divided over two aliquots and dried. One aliquot was resuspended with 800 μL ultrapure water (Sigma) and then distributed over four aliquots. Each sub-aliquot containing 200 μL PM suspension was used for one OP analysis.

We selected two acellular methods to evaluate oxidative potential: electron spin resonance (OP^ESR^) and dithiothreitol (OP^DTT^). Our application of these methods has been described in detail by Janssen et al. (2014). The rate of DTT consumption (expressed as nanomoles DTT/minute divided by sampled volume) was determined by linear regression of the remaining amount of DTT against time, based on two duplicate measurements. The ESR method is based on the trapping of PM-induced hydroxyl radicals (•OH) mainly generated via Fenton-type reactions in the presence of H_2_O_2_. 5,5-Dimethyl-1-pyrroline-N-oxide (DMPO) was used as the spin trap. OP^ESR^ was calculated as the average of the total amplitudes of the DMPO–OH quartet in arbitrary units (A.U.), divided by sampled volume.

No field blanks or duplicates were collected for PM_2.5_. However, for quality assurance, we analyzed 11 PM_10_ (PM ≤ 10 μm) field blanks. These were assumed to be representative of PM_2.5_ measurements because the same filter type and impactors were used (Eeftens et al. 2012b). All OP analyses were done in January 2014.

*Data analysis of spatial variation*. Descriptive statistics of the adjusted annual averages were calculated and stratified by site type. To assess the amount of spatial variation, the range (minimum–maximum) was calculated as a percentage of the mean. Analysis of variance (SAS version 9.3, PROC GLM; SAS Institute Inc.) was used to test for significant differences between the three site types. Ratios between site types were obtained by exponentiation of the slopes from a regression model with natural log (concentrations) as the dependent variable and site type as the independent variable. We assessed the specificity of the spatial OP pattern by calculating the correlations (*R*^2^) between both OP methods, and of each OP method with NO_2_, NO_x_, PM_2.5_ mass concentration, PM_2.5_ absorbance, and PM_2.5_ elemental composition as measured by XRF.

*LUR model development*. The LUR modeling procedure and description of the input data have been described in detail by Eeftens et al. (2012a). Briefly, potential predictor variables used for LUR model development were derived from GIS (ArcGIS; ESRI). In addition, the regional OP background estimate was offered as a predictor. The OP background was calculated by inverse distance squared weighted interpolation of OP concentrations measured at the regional sites, except the site itself. See Supplemental Material, Table S1, for an overview of the predictor variables and buffer sizes used to develop the LUR models. Predictor variables where many monitoring sites (*n* > 30) had zero values were excluded.

LUR models for OP^ESR^ and OP^DTT^ were developed following the standardized ESCAPE approach. Briefly, predictors yielding the highest adjusted *R*^2^ were subsequently added to the model if they conformed to the direction of effect defined *a priori* and added > 0.01 to the adjusted *R*^2^. The final models were checked for *p*-value (all predictors with *p* > 0.10 were excluded), co-linearity [variables with variance inflation factor (VIF) > 3 were removed and the model was rerun], influential observations (models with Cook’s D > 1 were further examined), and autocorrelation in the residuals (Moran’s *I*). We used two approaches to evaluate the final model: *a*) leave-one-out cross validation (LOOCV), which consecutively leaves out one site from the training data set and estimates model based on the remaining N-1 sites, leaving the model structure constant. The model predictions are then compared with measured values; *b*) holdout validation (HV), where we randomly select 10 training data sets stratified by site type (i.e., 50% of each site type, resulting in 20 sites), and develop new models based on these 20 sites. These new models are consecutively validated against the remaining 10 test data sets (Wang et al. 2012).

To evaluate whether LUR models for OP potentially have added value in epidemiological studies over the existing models in the ESCAPE study (see Supplemental Material, Table S2), we assessed the correlations between the OP model predictions and the previously developed LUR model predictions at 40 sites where only NO_2_ was measured. These sites were not used in OP and PM model development, but did have the same GIS predictor variables available. The LUR models for eight selected elements [copper (Cu), iron (Fe), potassium (K), nickel (Ni), vanadium (V), sulfur (S), silicon (Si), and zinc (Zn)] are available in de Hoogh et al. (2013) and PM_2.5_ models in Eeftens et al. (2012a). We used the NO_2_/NO_x_ models developed on the 40 PM_2.5_ sites (Wang et al. 2013).

## Results

*Quality control*. All OP^ESR^ and OP^DTT^ measurements were corrected with their corresponding mean field blank measurements (OP^ESR^: 850 A.U./m^3^; OP^DTT^: 0.12 nmol DTT/m^3^). For OP^DTT^, only three filter samples were below the limit of detection (LOD), whereas for OP^ESR^, one sample was below the LOD. All values were retained.

The correlations between mean concentrations based on measured values at 40 monitoring sites and mean concentrations after temporal adjustment were high for OP^ESR^ (*R*^2^ = 0.65) and moderate for OP^DTT^ (*R*^2^ = 0.46).

**Figure 1:**
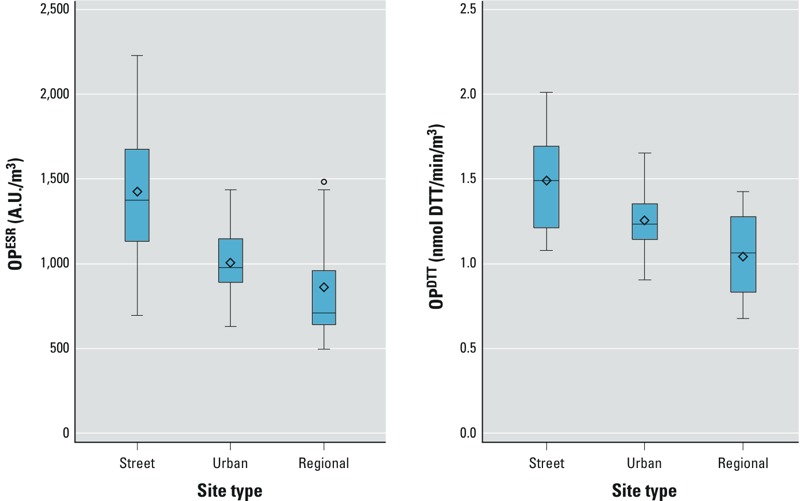
Adjusted annual average of OP^ESR^ (left) and OP^DTT^ (right) by site type. Median, mean, and 25th and 75th percentiles are shown in the box, whiskers indicate minimum and maximum values, and individual outliers are shown as points. *n* = 40 sites.

*Spatial variation*. Spatial variations and descriptive statistics of the average concentrations for OP^DTT^ and OP^ESR^ are shown in Figure 1 (see also Supplemental Material, Table S3). Annual mean levels for OP^DTT^ and OP^ESR^ showed substantial variation between site types. We also observed large variations within different site types. The spatial contrast (range, 102% of the mean) was lower for OP^DTT^ than for OP^ESR^ (range, 150% of the mean). Both OP^ESR^ and OP^DTT^ were consistently higher at the street sites. The mean street/urban background (S/UB) ratios were 1.2 for OP^DTT^ and 1.4 for OP^ESR^ and statistically significant (*p* < 0.05) for both (Table 1). Average regional/urban background (RB/UB) ratios were 0.8 for both OP^DTT^ and OP^ESR^, but only significant for OP^DTT^. We observed no distinctive regional patterns for either OP measurement (data not shown).

**Table 1:** Ratios between regional background (RB), urban background (UB), and street sites (S).

Component	S/UB	RB/UB	S/RB
OP^DTT^	1.2*	0.8*	1.4*
OP^ESR^	1.4**	0.8	1.7**
PM_2.5_	1.1**	1.0	1.2**
PM_2.5 _absorbance	1.5**	0.8*	1.8**
NO_2_	1.4**	0.7**	2.1**
NO_x_	1.7**	0.6**	2.7**
Fe	1.8**	0.7*	2.5**
Cu	1.7**	0.7**	2.5**
K	1.1	1.0	1.1
Ni	1.0	0.8	1.3
S	1.0	1.0	1.0
Si	1.6**	1.1	1.5**
V	1.0	0.8	1.2
Zn	1.1	0.9	1.1
*p < 0.05. **p <0.01.			

**Figure 2:**
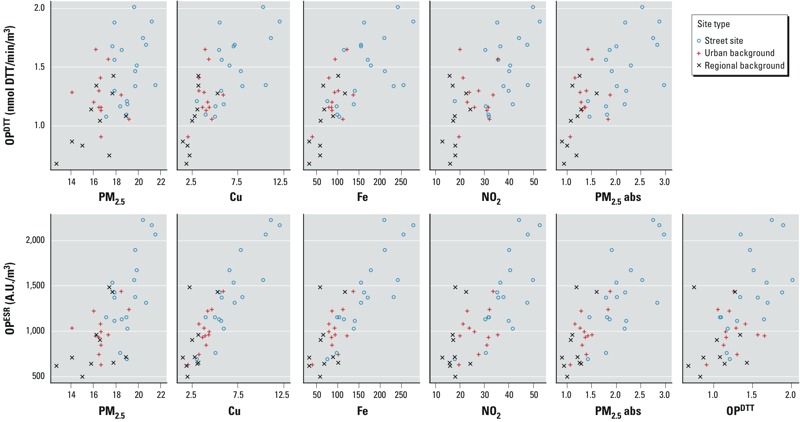
Relationship among measured annual average of OP^DTT^, OP^ESR^, Cu (ng/m^3^), Fe (ng/m^3^), NO_2_ (μg/m^3^), PM_2.5_ mass concentration (μg/m^3^), and PM_2.5_ absorbance (abs; 10^–5^/m) by site type; *n* = 40. The correlation coefficients (*R*^2^) are presented in Table 2.

*Correlations between measured OP and PM_2.5_ composition*. We found moderate correlations between OP^ESR^ and OP^DTT^ (Figure 2, Table 2; *R*^2^ = 0.35), and between both OP^ESR^ and OP^DTT^ and PM_2.5_ (*R*^2^ = 0.48 and 0.31, respectively). For OP^ESR^, the highest correlations were observed with the transition metals Cu and Fe (*R*^2^ = 0.76 and 0.71, respectively), whereas very low correlations were observed with K, S, Ni, V, and Zn (*R*^2^ < 0.20). As seen in Figure 2, we observed the highest correlations between OP^ESR^ and traffic markers (e.g., Fe, Cu, NO_2_, PM_2.5_ absorbance).

**Table 2:** Squared Pearson’s correlations (*R*^2^) of measured OP^ESR^, OP^DTT^ with PM_2.5_, PM_2.5_ absorbance, NO_2_, NO_x_, and eight selected PM_2.5_ elements.

Component	OP^ESR^	OP^DTT^
OP^DTT^	0.35	
PM_2.5_	0.48	0.31
PM_2.5 _absorbance	0.63	0.48
NO_2_	0.56	0.43
NO_x_	0.57	0.48
Cu	0.76	0.52
Fe	0.71	0.54
K	0.19	0.09
Ni	0.14	0.06
S	0.11	0.36
Si	0.39	0.21
V	0.04	0.04
Zn	0.05	0.24

The correlations between OP^DTT^ and PM_2.5_ components were generally lower than for OP^ESR^ (Table 2). OP^DTT^ also correlated highest with traffic markers (*R*^2^ = 0.43–0.54); however, these correlations were lower than for OP^ESR^. OP^DTT^ correlated poorly with K, Ni, and V (*R*^2^ < 0.10).

*Land use regression modeling*. For OP^DTT^, the regional background and road length (in a 500-m buffer) explained the largest contrast (Table 3), both resulting in an increased 0.3 OP units for a difference between the 10th and 90th percentile of the predictor. The model *R*^2^ value was 0.60, the LOOCV *R*^2^ was 0.47, and the HV *R*^2^ was 0.30 ± 0.08. Removing the regional OP^DTT^ from the final model reduced the *R*^2^ to 0.44.

**Table 3:** Description of developed LUR model for OP^DTT^.

Predictor	Regression coefficient^*a*^	Standard error	Pr > |t|	Partial *R*^2^
Intercept	0.08	0.26	0.76	
Regional estimate OP^DTT^	0.33	0.09	0.00	0.20
Road length 500 m buffer	0.31	0.09	0.00	0.49
Product T.I. and inverse distance^*b*^	0.15	0.05	0.00	0.53
Seminatural and forested area, 1,000 m	–0.11	0.06	0.095	0.55
^***a***^Regression slopes (see Supplemental Material, Table S2) multiplied by the difference between the 10th and 90th percentile for each of the predictors (0.43, 12997, 2214, 397834); intercept derives directly from model. Model *R*^2^ = 0.60; LOOCV *R*^2^ = 0.47; RMSE (root mean squared error) = 0.23 (nmol DTT/min/m^3^), HV *R*^2^ = 0.30 ± 0.08 (mean ± SE). *n* = 40 sites. ^***b***^Product of inverse distance to the nearest major road and the traffic intensity (T.I.) on this road (vehicles day^–1^ m^–1^).				

For the OP^ESR^ model, traffic load in a 50-m buffer explained the largest contrast (Table 4). Traffic load is the sum of the product of intensity and length of all road segments within a buffer. Traffic load incorporates all roads in a buffer, whereas the inverse distance–weighted traffic intensity variable included in the OP^DTT^ model involves a single road (nearest major road). The road length variable of the OP^DTT^ model does not incorporate traffic intensity. The OP^ESR^ model *R*^2^ value was 0.67, the LOOCV *R*^2^ was 0.60, and the HV *R*^2^ was 0.45 ± 0.17. Removing the regional OP^ESR^ from the final model reduced the *R*^2^ to 0.58.

**Table 4:** Description of developed LUR model for OP^ESR^.

Predictor	Regression coefficient^*a*^	Standard error	Pr > |t|	Partial *R*^2^
Intercept	327	177	0.07
Regional estimate OP^ESR^	434	142	0.00	0.19
Traffic load within 50 m	587	108	< 0.00	0.58
Population density within 5,000 m	305	115	0.01	0.64
^***a***^Regression slopes (see Supplemental Material, Table S2) multiplied by the difference between the 10th and 90th percentile for each of the predictors (764, 2890943, 375645); intercept derives directly from model. Model *R*^2^ = 0.67; LOOCV *R*^2^ = 0.60; RMSE = 280 (A.U./m^3^); HV *R*^2^ = 0.45 ± 0.17 (mean ± SE). *n* = 40 sites.				

Moran’s *I* tests to evaluate the spatial autocorrelation in the residuals was near zero and nonsignificant (*p* > 0.05) for both OP models.

*Correlation between model-predicted OP and PM characteristics*. We found moderate correlations (Table 5; *R*^2^ = 0.44) between OP^DTT^ and OP^ESR^ model predictions. OP^DTT^ and OP^ESR^ model predictions were moderately to highly correlated (*R*^2^ = 0.37 and 0.52, respectively) with PM_2.5_ model predictions. OP^DTT^ model predictions were highly correlated with PM_2.5_ absorbance (*R*^2^ = 0.50) and NO_2_ model predictions (*R*^2^ = 0.54). The correlations between OP^DTT^ model predictions and majority of the components were mostly moderate (*R*^2^ = 0.31–0.49), except for V, Ni, and Zn (*R*^2^ = 0.09–0.22). OP^ESR^ model predictions were generally highly correlated (*R*^2^ = 0.50–0.84) with the majority of the components, except for K, Ni, V, and Zn model predictions (*R*^2^ = 0.07–0.33). The highest correlations were found between model predictions of OP^ESR^ and Cu (*R*^2^ = 0.79) and Fe (*R*^2^ = 0.84). The correlations between model predictions at 40 sites not used for the modeling were generally similar to those between the measurements.

**Table 5:** Squared Pearson’s correlations (*R*^2^) of LUR models predictions for OP^ESR^, OP^DTT^ with PM_2.5_, PM_2.5_ absorbance, Cu, Fe, S, Si, NO_2_, and NO_x_ at 40 sites not used in modeling.

Component	OP^ESR^	OP^DTT^
OP^DTT^	0.44
PM_2.5_	0.52	0.37
PM_2.5_ absorbance	0.65	0.50
NO_2_	0.56	0.54
NO_x_	0.50	0.42
Cu	0.79	0.46
Fe	0.84	0.49
K	0.25	0.32
Ni	0.33	0.09
S	0.52	0.36
Si	0.55	0.31
V	0.33	0.10
Zn	0.07	0.22

## Discussion

We found substantial spatial variation for both OP^ESR^ and OP^DTT^, with higher contrasts for OP^ESR^ than OP^DTT^. OP^ESR^ was moderately correlated with OP^DTT^ and PM_2.5_ mass concentrations, but highly correlated with PM_2.5_ absorbance, NO_2_/NO_x_, and especially the transition metals Fe and Cu. In comparison, these correlations were lower for OP^DTT^. The LUR model for OP^DTT^ had an explained variance of 60%, whereas the LUR models for OP^ESR^ had an explained variance of 67%. The LUR model performance was better for the OP^ESR^ model (LOOCV *R*^2^: 0.60; HV *R*^2^: 0.45) than for OP^DTT^ (LOOCV *R*^2^: 0.47; HV *R*^2^: 0.30).

*Spatial contrasts*. Although studies have evaluated the spatial contrasts of OP for different site types, none has characterized the spatial contrast in such an extensive way as in this study with 40 sites. Consistent with the results from our study, OP^ESR^ was generally higher at sites dominated by traffic (Boogaard et al. 2011; Janssen et al. 2014; Shi et al. 2006; Wessels et al. 2010). Other studies in the Netherlands also found higher OP^ESR^ at street sites than at the urban background site (Boogaard et al. 2011; Janssen et al. 2014). Janssen et al. (2014) found that OP^ESR^ of PM_2.5_ was 1.1 higher at a “stop&go” site, and 5.1 higher at a continuous traffic site than the urban background site. Boogaard et al. (2011) found a median ratio of 3.6 between street and corresponding urban background site, where the ratios ranged from 1.6 to 6.8, depending on the street configuration in a study of eight busy streets. In the study by Boogaard et al. (2011) OP^ESR^ of PM_10_ was measured, which could explain the higher contrast documented therein compared with our study, because the transition metals (Fe, Cu) to which ESR primarily responds are abundant in the coarse fraction of PM.

The S/UB contrast was lower for OP^ESR^ (ratio of 1.4) than for the transition metals Fe and Cu (Table 1; ratio of 1.8 and 1.7, respectively). In our previous study of the temporal and spatial variation of OP^ESR^ for 11 National Air Quality Monitoring sites in the Netherlands, we also found a lower contrast for OP^ESR^ than for Fe and Cu (Yang et al. 2015). Janssen et al. (2014) also reported lower S/UB ratios for OP^ESR^ (1.1 and 5.1) than for Fe (ratios of 2.2 and 6.7) and Cu (ratios of 1.8 and 6.0) at two different street sites. This could potentially be due to the methods used to analyze the chemical composition (energy dispersive XRF and inductively coupled plasma mass spectrometry) in the aforementioned studies, because only the total metal content was measured. As a result, the multiple valence states of the transition metals were not fully captured. Measurement of OP^ESR^ is based on the Fenton reaction between peroxides and transition metals, which leads to the production of hydroxyl radicals, and is dependent not only on the valence state of the metal, but also on solubility (Valavanidis et al. 2005). Previous studies have shown that certain transition metal ions [Fe(II), Cu(I)] have a higher capability to generate hydroxyl radicals than others [Fe(III)] (Shi et al. 2003; Valavanidis et al. 2005). Therefore, one would not expect a perfect agreement between total transition metal and OP^ESR^.

The S/UB contrast was higher for both OP methods than for the PM_2.5_ mass concentration (ratio = 1.1), but lower than for PM_2.5_ absorbance (Table 1; ratio = 1.5), NO_2_ (ratio = 1.4), and NO_x_ (ratio = 1.7). The higher spatial contrast for OP compared to PM_2.5_ is consistent with previous studies (Boogaard et al. 2011; Janssen et al. 2014). The lower spatial contrast compared with PM_2.5_ absorbance and NO_2_/NO_x_ could be attributable to the relatively larger influence of other sources than local traffic on oxidative potential.

The lower S/UB ratio for OP^DTT^ than for OP^ESR^ is consistent with observations at two traffic sites in the study by Janssen et al. (2014) (e.g., ratio of 2.4 for OP^DTT^ and ratio of 5.1 for OP^ESR^). These differences can be attributed to the different components in the PM mixture to which OP^ESR^ and OP^DTT^ are sensitive. Although OP^ESR^ is especially sensitive to transition metals driving •OH generation mechanisms via the Fenton reaction, OP^DTT^ is associated with organic compounds such as polycyclic aromatic hydrocarbons (PAH) and organic carbon (OC), and to a certain degree transition metals (Charrier and Anastasio 2012; Li et al. 2003). Jedynska et al. (2014) assessed the contrasts of PAHs and OC for 16 sites of the 40 sites in our study area and found lower contrasts for OC (S/UB = 1.05) than for OP^DTT^ (S/UB = 1.21, derived from this study using the 16 sites). In contrast, the ratio of PAHs (S/UB = 1.88) was higher (Jedynska et al. 2014). The OC contrast (associated with OP^DTT^) was lower than the contrast in transition metals (associated with OP^ESR^), which could explain the higher OP^ESR^ contrast than OP^DTT^. PAH contrasts were similar to transition metals contrasts (Jedynska et al. 2014).

*Correlations between measured OP and PM characteristics*. Consistent with previous studies, we found high spatial correlations between OP^ESR^ and all traffic-related PM components Fe, Cu, and PM_2.5_ absorbance (Boogaard et al. 2011; Künzli et al. 2006). The high correlations between transition metals and OP^ESR^ (Table 2; Cu: *R*^2^ = 0.76; Fe: *R*^2^ = 0.71) are comparable with those from Boogaard et al. (2011), who analyzed OP^ESR^ of PM_10_ (Pearson’s *R* ≥ 0.95 for Cu and Fe). In comparison, Künzli et al. (2006) found much lower correlations (Spearman’s *r* = 0.39 for Cu, *r* = 0.45 for Fe), possibly because OP^ESR^ was analyzed at 20 urban background sites only. As seen in Figure 2, the high correlations are largely driven by the street sites (*n* = 18) and suggest a direct impact of these transition metals on OP.

Saffari et al. (2014) assessed the seasonal and spatial variation of OP^DTT^ for quasi-ultrafine particles (PM_0.25_) at 10 locations across the Los Angeles Basin, California, and found across seasons the highest correlations between DTT activity and carbonaceous PM (Pearson’s *R* > 0.70 for OC, both soluble and insoluble). Correlations between OP^DTT^ and PM composition varied depending on the season, but are comparable with our results that were adjusted for temporal variation. However, they also found high correlations between the transition metals (e.g., Fe, Cu, V, Zn, Cr) and the organic compounds, especially for the quasi-ultrafine range, where the common source is vehicular emissions (Saffari et al. 2014). Nevertheless, for DTT, we found similar correlations with all inorganic traffic markers, which indicate no direct impact of one specific component on OP^DTT^ and thus is a less traffic-specific measure than OP^ESR^.

*Performance of the LUR models*. To our knowledge, only one study has developed a model for outdoor OP, but their modeling approach differs from ours on several accounts. Yanosky et al. (2012) modeled OP of PM_10_ (in OP per μg PM_10_) for greater London, where OP was measured as the depletion rate of GSH (OP^GSH^). The model used weekly averages from the year 2002 through 2006 and a geostatistical spatiotemporal model was developed, with an *R*^2^ of 0.52 (cross-validation *R*^2^ = 0.44). The two predictors of spatial variation in OP^GSH^ were brake and tire wear emissions of PM_10_ from local traffic (within 50 m) and NO_x_ from heavy-duty vehicles with a negative slope.

We developed LUR models for OP with reasonably good explained variance that was slightly higher for the OP^ESR^ model (*R*^2^ = 0.67) than for the OP^DTT^ model (*R*^2^ = 0.60). This might be attributable to the larger impact of local traffic on OP^ESR^ compared with OP^DTT^ as documented by the measurements. LUR models can effectively model traffic effects in our study, due to adequate representation of traffic sites in the ESCAPE study and good availability of traffic predictors compared to other sources (e.g., wood burning). Both models contained large buffer variables for urbanity, consistent with the 25% higher measured OP values at the urban versus regional background sites. Both models included a regional estimate, which accounts for the regional contrast in background concentrations, because other predictor variables could not explain the large-scale spatial trends in our study area. We included the regional background OP in the model instead of subtracting it from all measurements to allow assessment of the contribution of regional background to the overall variability in OP. Exclusion of the regional estimate, which explained 19–20% of the variance for both OP methods, led to a more substantial reduction of explained variance for the OP^DTT^ model (15.4%) than for OP^ESR^ (9%).

The differences between modeled *R*^2^ and HV *R*^2^ for both OP models in our study are comparable with findings from another study in the Netherlands that evaluated the performance of NO_2_ and PM_2.5_ absorbance (using 20 training sites). A difference of 27% between modeled *R*^2^ and HV *R*^2^ was found for NO_2_, whereas a difference of 16% was found for PM_2.5_ absorbance (Wang et al. 2013). The HV procedure we applied might have resulted in too low *R*^2^ values because the training sets included only 20 sites, which likely resulted in less robust models than the developed models that were based on 40 sites. Especially for OP^ESR^, an HV *R*^2^ of 45% (± 17%) is in the range of those previously reported by Wang et al. (2013). In another study by Wang et al. (2012), the difference between model *R*^2^ and HV *R*^2^ for NO_2_ was 27% for 24 sites and 18% for 48 sites. The gap between model and HV *R*^2^ likely reflects modest overfitting (Wang et al. 2012, 2013). Our LUR models thus performed similarly to models developed for more often modeled pollutants, including NO_2_.

*Comparison of OP LUR models with other ESCAPE LUR models*. Several LUR models (see Supplemental Material, Table S2) were developed in the ESCAPE project and used for cohort exposure assessment. Although the performance of the LUR models for both OP^ESR^ and OP^DTT^ was comparable to the PM_2.5_ model (*R*^2^ = 0.67), it was lower than the models of traffic-related components such as Fe, Cu, NO_x_/NO_2_, and PM_2.5_ absorbance (see Supplemental Material, Table S2; *R*^2^ = 0.78–0.92). OP is probably less affected by local traffic than absorbance or Cu, as indicated by the lower measured S/UB concentration ratios for OP. Furthermore, OP is an indicator of PM-induced oxidative stress, and we have no specific predictor variables for the biological activity. Despite the inclusion of similar (traffic) predictors in the OP and other models, the relative importance of predictors may differ in the OP model versus models for other pollutants. Dispersion models are not feasible because specific emission factors for OP are not available.

An important issue to be considered is the added value of the OP models for application in epidemiological studies compared with the existing models, which can well predict variation of traffic-related components. Furthermore, when applying the models to addresses of subjects in cohort studies, it is imperative that the predictions of the OP models can be disentangled not only from each other, but also from the existing models for PM_2.5_ mass concentration, PM_2.5_ absorbance, and nitrogen oxides. The moderately high correlations between OP model predictions and PM_2.5_ mass concentration predictions suggest some potential to evaluate whether OP predicts health effect better than the regulated metric PM_2.5_.

The OP^ESR^ model predictions were generally highly correlated with predictions of most traffic-related elements (*R*^2^ > 0.50), especially with Cu. Similar to the OP^ESR^ model, traffic- and road-related variables were the most important predictors for these models (see Supplemental Material, Table S2). This suggests it might be difficult to separate the effects of OP^ESR^ from the existing models of traffic components in the Netherlands. Nevertheless, OP^ESR^ could still be important in epidemiological studies because it might provide more consistent effect estimates in multiple countries, if the assumption of higher biological relevance compared with total metal concentrations is correct. Despite the high correlations between OP^ESR^ and elements such as Fe and Cu, the absolute concentration ratios may differ due to the difference in biological availability between countries.

The OP^DTT^ model predictions were moderately correlated with predictions of most elements (*R*^2^ < 0.50), except for PM_2.5_ absorbance and NO_2_. This indicates that it should be possible to distinguish between the independent effects of OP^DTT^ and PM_2.5_ components in epidemiological studies. Finally, the moderate correlation (*R*^2^ = 0.44) between the predictions of the OP^DTT^ and the OP^ESR^ model suggests it might be possible to investigate which of the two OP assays predicts health effects better. Alternatively, because the two assays respond to different PM components, we can evaluate whether OP^DTT^ and OP^ESR^ together predict health effects better than PM_2.5_ mass concentration.

## Conclusion

LUR models explained a large fraction of the spatial variation of the two OP metrics. The moderate correlations among the predictions of OP^DTT^, OP^ESR^, and PM_2.5_ models offer the potential to investigate which metric is the strongest predictor of health effects.

## Supplemental Material

(777 KB) PDF
