# Supplementary material for: Spatial Variation and Land Use Regression Modeling of the Oxidative Potential of Fine Particles
Source: Environ Health Perspect. 2015 Apr 3;123(11):1187–92. doi: 10.1289/ehp.1408916 (PMC4629740; doi:10.1289/ehp.1408916)
Supplement: (777 KB) PDF [file ehp.1408916.s001.acco.pdf]

Environ Health Perspect
doi: 10.1289/ehp.1408916

Note to Readers: *EHP* strives to ensure that all journal content is accessible to all readers. However, some figures and Supplemental Material published in *EHP* articles may not conform to 508 standards due to the complexity of the information being presented. If you need assistance accessing journal content, please contact ehp508@niehs.nih.gov. Our staff will work with you to assess and meet your accessibility needs within 3 working days.

Supplemental Material

Spatial Variation and Land Use Regression Modeling of the Oxidative Potential of Fine Particles

Aileen Yang, Meng Wang, Marloes Eeftens, Rob Beelen, Evi Dons, Daan L.A.C. Leseman, Bert Brunekreef, Flemming R. Cassee, Nicole A.H. Janssen, and Gerard Hoek

Table of Contents

Figure S1. Overview of the sampling sites in study area. The study area is characterized by minor altitude differences and an overall high population density. Ten regional background monitoring sites were selected in small villages and countryside settings, to capture regional differences resulting from long-range transport. Five sites were selected in the larger cities of AMSTERDAM, Rotterdam, Utrecht, and Antwerp (300,000-800,000 inhabitants), while in the smaller cities of Amersfoort, Groningen, Doetinchem, and Maastricht (50,000-200,000 inhabitants) only three or two sites were selected. Major sea ports are present near both Rotterdam and Antwerp. N=40 sites.

Description of the sampling site selections

Table S1. Predictor variables, direction and buffer sizes considered for development of LUR models.

Table S2. Description of previously developed LUR models for the Netherlands/Belgium study area in the framework of ESCAPE.

Table S3. Descriptive statistics and overall contrasts of adjusted annual average concentrations of OP^{DTT} (nmol DTT/min/m^3), OP^{ESR} (A.U./m^3) by site type.

References

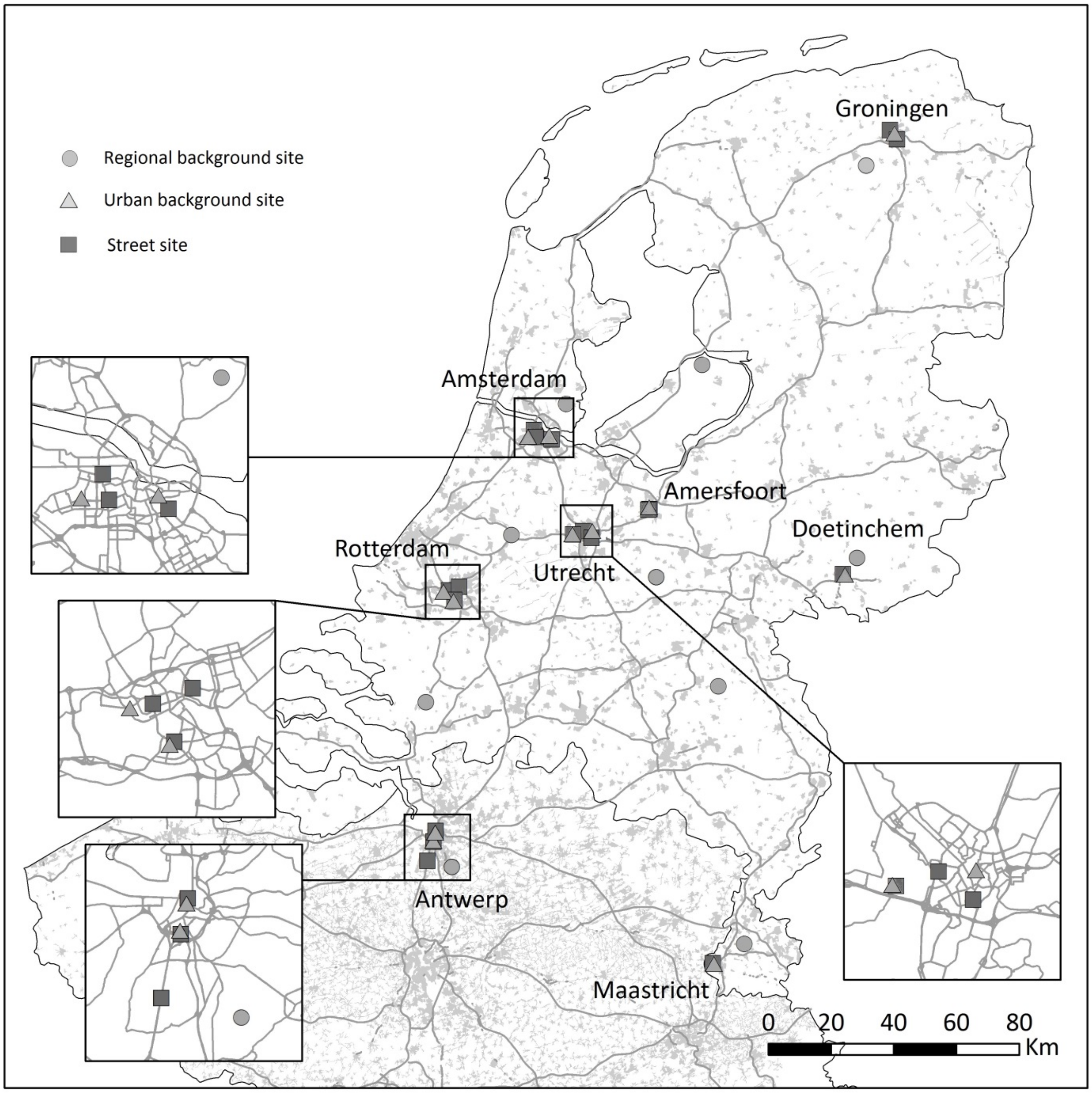

Figure S1. Overview of the sampling sites in study area. The study area is characterized by minor altitude differences and an overall high population density. Ten regional background monitoring sites were selected in small villages and countryside settings, to capture regional differences resulting from long-range transport. Five sites were selected in the larger cities of Amsterdam, Rotterdam, Utrecht, and Antwerp (300,000-800,000 inhabitants), while in the smaller cities of Amersfoort, Groningen, Doetinchem, and Maastricht (50,000-200,000 inhabitants) only three or two sites were selected. Major sea ports are present near both Rotterdam and Antwerp. N=40 sites.

Description of the sampling site selections

To cover the geographical spread of five cohort studies, the study areas of the Netherlands and Belgium comprised a large geographical area, involving eight major cities. These two areas were combined and treated as one study area to comprise a total of 40 monitoring sites. All sampling sites were selected locally by considering the spatial distribution of the cohorts of interest and the specific characteristics of the study area. Measurement sites were selected to reflect a large diversity of potential sources of air pollution variability such as population density, traffic intensity, industry, proximity to harbors etc.

Ten regional background monitoring sites were selected in small villages and countryside settings, to capture regional differences resulting from long-range transport. Five sites were selected in the larger cities of Amsterdam, Rotterdam, Utrecht, and Antwerp (300,000-800,000 inhabitants), while in the smaller cities of Amersfoort, Groningen, Doetinchem, and Maastricht (50,000-200,000 inhabitants) only three or four sites were selected. Both urban background and street sites were sampled in each of these towns. A variety of street sites with different traffic intensity, distance of the sampling site to the road, and different street configurations was included.

The Dutch & Belgian study area is characterized by minor altitude differences and an overall high population density. Major sea ports are present near both Rotterdam and Antwerp.

Table S1. Predictor variables, direction and buffer sizes considered for development of LUR models.

Predictor variable	Variable name	Buffer (m)	Direction[a]
Coordinates (X,Y)	XCOORD, YCOORD		1
Industry	INDUSTRY	1000, 5000	1
Port	PORT	5000	1
Urban green	URBGREEN	1000, 5000	-1
Semi-natural and forested areas	NATURAL	1000, 5000	-1
Sum of URBGREEN&NATURAL	UGNL	500, 1000, 5000	-1
Population data on a European level	POPEEA	100, 300, 500, 1000, 5000	1
Regional estimates, based on interpolated inverse distance squared weighting of concentrations measured at regional sites, except the site itself	REG_EST_opdtt, REG_EST_opesr		1
Sum of LDRES (low density residential land) and HDRES (high density residential land)	HDLDRES	50, 100, 300, 500, 1000	1
Traffic intensity on nearest road	TRAFNEAR		1
Distance to nearest road (local road network)	DISTINVNEAR1, DISTINVNEAR2		1
Product of traffic intensity on nearest road&inverse distance to nearest road and distance squared (local road network)	INTINVDIST, INTINVDIST2		1
Traffic intensity on nearest major road	TRAFMAJOR		1
Inverse distance and inverse distance squared to nearest major road (local road network)	DISTINVMAJOR1, DISTINVMAJOR2		1
Product of traffic intensity on nearest major road & inverse of distance to the nearest major road and distance squared	INTMAJORINVDIST, INTMAJORINVDIST2		1
Total traffic load of major roads in a buffer (sum of (traffic intensity * length of all segments))	TRAFMAJORLOAD	50, 100, 300, 500, 1000	1
Total traffic load of roads in a buffer (sum of (traffic intensity * length of all segments))	TRAFLOAD	50, 100, 300, 500, 1000	1
Heavy-duty traffic intensity on nearest road	HEAVYTRAFNEAR		1
Product of heavy-duty traffic intensity on nearest road and inverse of distance to the nearest road and distance squared	HEAVYINTINVDIST, HEAVYINTINVDIST2		1
Heavy-duty traffic intensity on nearest major road	HEAVYTRAFMAJOR		1
Total heavy-duty traffic load of major roads in a buffer (sum of (heavy-duty traffic intensity * length of all segments)	HEAVYTRAFMAJORLOAD	50, 100, 300, 500, 1000	1
Total heavy-duty traffic load of all roads in a buffer (sum of (heavy-duty traffic intensity * length of all segments))	HEAVYTRAFLOAD	50, 100, 300, 500, 1000	1
Road length of all roads in a buffer	ROADLENGTH	50, 100, 300, 500, 1000	1
Road length of all major roads in a buffer	MAJORROADLENGTH	50, 100, 300, 500, 1000	1
Inverse distance and inverse squared distance to nearest road (central road network)	DISTINVNEARC1, DISTINVNEARC2		1
Inverse distance and inverse squared distance to nearest major road (central road network)	DISTINVMAJORC1, DISTINVMAJORC2		1

[a]Predefined direction of effect, negative for green space and natural areas.

Table S2. Description of previously developed LUR models for the Netherlands/Belgium study area in the framework of ESCAPE.

Exposure	Model R^2	LOOCV R^2	LUR model[a]	Source
OP^{ESR}	0.67	0.60	326.53554 +0.56805*REG_EST_opesr +2.0309E-4*TRAFLOAD_50+8.1288E-4*POPEEA_5000	Present study
OP^{DTT}	0.60	0.47	0.08096 +0.76684*REG_EST_opdtt+2.364E-5*ROADLENGTH500 +6.977E-05*INTMAJORINVDIST -2.65222E-07*NATURAL_1000;	Present study
$PM_{2.5}$	0.67	0.61	9.46 +0.42*REG_EST_PM25 +0.01*MAJORROADLENGTH50+ 2.28E-09*TRAFMAJORLOAD_1000	(Eeftens et al. 2012)
$PM_{2.5}$ abs	0.92	0.89	0.07 +2.95E-09*TRAFLOAD_500 + 2.93E-03*MAJORROADLENGTH50+0.85*REG_EST_PM25abs +7.90E-09*HDLDRES_5000+1.72E-06*HEAVYTRAFLOAD_50	(Eeftens et al. 2012)
NO_2	0.88	0.80	41.11+1.90E-7*TRAFLOAD_500+0.099*MAJORROADLENGTH50+8.65E-5*HEAVYTRAFLOAD_50+ 6.43E-7*PORT_5000+2.35E-7*HEAVYTRAFMAJORLOAD_1000-9.8E-5*XCOORD	(Wang et al. 2013)
NOx	0.91	0.82	3.25+0.74*REG_EST_NOx+4.22E-6*TRAFLOAD_50+6.36E-4*POPEEA_1000 +2.39e-6*HEAVYTRAFLOAD_500+71.65*DISTINVMAJOR1+0.21*MAJORROADLENGTH25	(Wang et al. 2013)
Cu	0.83	0.81	6.5 +4.80E-08*HDLDRES_5000 +5.00E-07*TRAFMAJORLOAD_50 + 1.00E-02*MAJORROADLENGTH50-6.70E-06*(XCOORD+YCOORD)	(de Hoogh et al. 2013)
Fe	0.78	0.73	149E+1.40E-06*HDLDRES_5000 +1.90E-03*TRAFNEAR+ 8.70E-06*TRAFMAJORLOAD_50 -1.50E-04*(XCOORD+YCOORD)	(de Hoogh et al. 2013)
S	0.32	0.27	1240 +1.10E-02*POPEEA_500-8.50E-04*YCOORD	(de Hoogh et al. 2013)
Si	0.46	0.39	146 +2.60E-03*TRAFNEAR-1.10E-04*(XCOORD+YCOORD)	(de Hoogh et al. 2013)
Ni	0.76	0.72	3.7+8.6E-8*PORT_5000 - 1.2E-5*XCOORD	(de Hoogh et al. 2013)
K	0.31	0.25	155+3.5E-7*TRAFMAJORLOAD_300+1.4E-4*(XCOORD-YCOORD)	(de Hoogh et al. 2013)
V	0.68	0.63	5.6+2.0E-7*PORT_5000 - 1.8E-5*XCOORD	(de Hoogh et al. 2013)

[a]See Table S1 for detailed description of variable names. Some variables are buffers with _X indicating the radius of the buffer in meters. Regional estimate (REG_EST_); port (PORT_X); natural land (NATURAL_X); the sum of high and low density residential land (HDLDRES_X); the sum of (traffic intensity * length of all road segments) within a buffer (vehicles day^{-1} m) for all roads (TRAFLOAD_X), for all major road segments (TRAFMAJORLOAD_X), for heavy traffic (HEAVYTRAFLOAD_X) and heavy traffic on major roads (HEAVYTRAFMAJORLOAD_X); population data on an European level (N) (POPEEA_X); total length (m) of all roads (ROADLENGTH_X) and all major road segments (MAJORROADLENGTH_X); traffic intensity on the nearest road (TRAFNEAR); X-coordinate (XCOORD); Y-coordinate (YCOORD); the product of inverse distance to the nearest major road and the traffic intensity on this major road (INTMAJORINVDIST); inverse distance (m^{-1}) to the nearest major road in the local network (DISTINVMAJOR1).

Table S3. Descriptive statistics and overall contrasts of adjusted annual average concentrations of OP^{DTT} (nmol DTT/min/m^3), OP^{ESR} (A.U./m^3) by site type.

Exposure	SITETYPE	Mean	StdDev	Median	min	p10	p25	p50	p75	p90	max	range/mean
OP^{DTT}	Regional	1.04	0.26	1.06	0.68	0.71	0.83	1.06	1.28	1.38	1.43	72%
	Street	1.49	0.29	1.49	1.08	1.10	1.21	1.49	1.69	1.89	2.01	63%
	Urban	1.26	0.21	1.23	0.91	1.06	1.14	1.23	1.35	1.57	1.65	59%
	All	1.31	0.31	1.28	0.68	0.87	1.1	1.28	1.55	1.74	2.01	102%
OP^{ESR}	Regional	861	343	711	496	556	641	711	960	1460	1484	115%
	Street	1426	448	1374	694	759	1134	1374	1674	2170	2228	108%
	Urban	1005	221	976	630	745	889	976	1149	1240	1437	80%
	All	1159	438	1095	496	642	781	1095	1434	1874	2228	150%

References

De Hoogh K, Wang M, Adam M, Badaloni C, Beelen R, Birk M, et al. 2013. Development of Land Use Regression Models for Particle Composition in Twenty Study Areas in Europe. Environ Sci Technol 47:5778–5786; doi:10.1021/es400156t.

Eeftens M, Beelen R, de Hoogh K, Bellander T, Cesaroni G, Cirach M, et al. 2012. Development of Land Use Regression Models for PM2.5, PM2.5 Absorbance, PM10 and PMcoarse in 20 European Study Areas; Results of the ESCAPE Project. Environ. Sci. Technol. 46:11195–11205; doi:10.1021/es301948k.

Wang M, Beelen R, Basagana X, Becker T, Cesaroni G, de Hoogh K, et al. 2013. Evaluation of Land Use Regression Models for NO2 and Particulate Matter in 20 European Study Areas: The ESCAPE Project. Environ. Sci. Technol. 47:4357–4364; doi:10.1021/es305129t.
